# Sports Medicine: Nutritional Sciences and Nutritional Biochemistry, Focusing on Nutritional Supplements from the FFNFO Framework to Contamination

**DOI:** 10.3390/life14111392

**Published:** 2024-10-29

**Authors:** Diego A. Bonilla, Jorge L. Petro, Richard B. Kreider, Roberto Cannataro

**Affiliations:** 1 Research Division, Dynamical Business & Science Society—DBSS International SAS, Bogotá 110311, Colombia; jlpetros@dbss.pro; 2 Hologenomiks Research Group, Department of Genetics, Physical Anthropology and Animal Physiology, University of the Basque Country (UPV/EHU), 48940 Leioa, Spain; 3 Research Group in Physical Activity, Sports and Health Sciences (GICAFS), Universidad de Córdoba, Monteria 230002, Colombia; 4 Exercise & Sport Nutrition Laboratory, Human Clinical Research Facility, Texas A&M University, College Station, TX 77843, USA; rbkreider@tamu.edu; 5 Department of Pharmacy, Health and Nutritional Sciences, University of Calabria, 87036 Rende, Italy

Sports medicine has become a well-established field with a wide range of applications, from competitive and professional athletics to general well-being. More specifically, nutrition has joined the many disciplines that are now of great public interest, thus creating an ever-increasing demand for an empirical and systematic approach towards its relationship with sports performance and health. Specifically, this Special Issue, entitled “Sports Medicine: Nutritional Sciences and Nutritional Biochemistry”, presents fresh insights into several key themes in sports nutrition and health tailored to various populations.

On the topic of burnout and diet, Morales-Suárez-Varela et al. investigated 183 young basketball players aged 8 to 15, finding that adherence to the Mediterranean diet was associated with lower burnout levels, particularly in those who spent less time on sedentary activities. This underscores the critical role of balanced nutrition in supporting young athletes’ mental and physical health. Shifting to eating behavior and physical activity, Fernandes and colleagues showed through structural equation modeling that higher physical activity levels lead to more self-determined eating behaviors. This, in turn, reduces the likelihood of external and emotional eating, emphasizing how exercise can positively shape eating habits.

In terms of public health and sports performance, Droste et al. conducted a two-year analysis across the top European soccer leagues, revealing a significant correlation between national COVID-19 incidence and case occurrences within leagues. Notably, La Liga players experienced the shortest time loss per COVID-19 case, highlighting league-specific variations in case management. Also in soccer players, Aguinaga-Ontoso and co-workers conducted a systematic review of nutritional interventions for athletic performance. They found that specific strategies, such as creatine monohydrate supplementation, bicarbonate solutions, and high-carb diets, significantly enhanced strength, endurance, and other performance metrics, though no dietary interventions were found to improve recovery. These findings highlight the importance of tailored nutrition strategies in optimizing athletic performance.

Finally, addressing injury prediction and body composition in team sports, Bertuccioli et al. explored the potential of bioimpedance vector analysis (BIVA) to predict injuries in professional basketball players. The study subjects included 14 male athletes who were part of the US Victoria Libertas Basketball Pesaro team that competes in the Italian Serie A championship, aged between 20 and 39 years. Following an analysis of training and matchday injuries, the authors noted several important correlations. They found that the number of training injuries was positively related to greater upper body reactance, whereas increased reactance in the right lower limb was associated with shorter injury duration. Furthermore, in match players, they found that the number of injuries sustained was negatively related to higher reactance in the right upper limb, perhaps indicating that body composition metrics can provide a better indicator of injury risk. Their findings suggest that BIVA might be a key method for the evaluation of injury risk and performance, hence there is a need for further studies to establish this.

Considering that no studies on dietary supplements were submitted to this Special Issue, we would like to highlight several key factors that we believe are important for the research community and practitioners. Nutritional supplements are commercially available products characterized by containing a nutrient or dietary ingredient formulated to complement the diet in populations with special needs or specific goals. These ingredients can include vitamins, amino acids and their derivatives, minerals, or glandular extracts, herbs, or other plants in commercial forms such as tablets, soft gels, gel capsules, liquids, or powders that are taken orally. In 1994, the United States approved the Dietary Supplement Health and Education Act (https://www.congress.gov/bill/103rd-congress/senate-bill/784, accessed on 29 July 2024), which classifies supplements in a special category of “foods”. In Europe, Directive 2002/46/EC from the European Parliament (https://eur-lex.europa.eu/eli/dir/2002/46/2024-02-06, accessed on 29 July 2024) established guidelines regarding food supplements.

Unfortunately, misinformation accelerated by influencers and YouTubers without training in human nutrition and unethical practices by certain brands, the use of low-quality raw materials to cut costs, and high commercial demand have created a landscape prone to various issues in the supplement industry, but even when valid, misuse could lead to considerable sides effects [1]. Sports supplements are now added to athletes’ regular diets to enhance energy availability during physical exertion, accelerate post-exercise recovery processes, and generally improve physical performance [2,3]. However, attention must also be paid to misinterpretation by nutrition professionals who apply classification systems designed exclusively for high-performance athletes (such as the ABCD system from the Australian Institute of Sport) to physically active individuals who attend fitness centers for esthetic or health reasons. In this regard, we encourage practitioners to use the ABC categorization system proposed by the International Society of Sports Nutrition (ISSN), which is also based on the quality and quantity of scientific support available for both the athletic and recreationally active population [3]. For tactical populations, the military community, healthcare providers, and Department of Defense civilians should consider and promote the “Operation Supplement Safety” program (https://www.opss.org/, accessed on 20 August 2024).

Recently, some authors have introduced the framework Food First but Not Always Food Only (FFNFO), which highlights seven special scenarios for the responsible use of supplements [4]:

- Some nutrients are difficult to obtain in sufficient amounts from the diet or may require excessive energy intake and/or the consumption of other nutrients.
- Some nutrients are abundant only in foods that athletes do not eat or do not like.
- The nutrient content of certain foods with established ergogenic benefits is highly variable.
- Concentrated doses of certain nutrients are needed to correct deficiencies and/or promote immune tolerance.
- Some foods may be difficult to consume before, during, or immediately after exercise.
- Supplements certified by third-party organizations (e.g., NSF, BSCG, Informed Sport, etc.) could help where there are concerns about food hygiene or contamination [4].

It has been shown that various supplements may contain contaminants that are unethically not disclosed on the nutrition label. This gives rise to the concept of contaminant supplementation, where there is a risk both to health and of positive doping results due to the inadvertent consumption of substances prohibited by the World Anti-Doping Agency [5]. Contamination can occur for several reasons, whether accidental or intentional, including (i) the presence of contaminants in the raw materials and a low-quality manufacturing process (such as the sharing of materials during production or inadequate cleaning), (ii) alterations during transportation (including packaging and storage), (iii) fraudulent labels indicating undisclosed ingredients or different quantities, and (iv) deliberate contamination to enhance efficacy [6].

Among the common contaminants reported in the scientific literature are anabolic androgenic steroids, sibutramine, clenbuterol, hormonal peptides, methylhexanamine, endocrine disruptors, and higenamine, along with various molecules that have not yet been chemically described [7,8]. It is important to highlight that the minimum required performance levels of these substances are extremely low, which increases the risk. For example, the limits vary between 0.4 and 2.5 ng/mL for anabolic androgenic steroids (https://www.wada-ama.org/sites/default/files/resources/files/td2022mrpl_v1.0_final_eng.pdf, accessed on 29 July 2024). These sources of accidental or intentional contamination in supplements are verified through quantitative chemical analysis using high-performance liquid chromatography coupled with mass spectrometry (HPLC-MS), which allows for the analysis of the athletes’ exposome [9]. Generally, products with a higher risk of contamination include fat burners, mass gainers, testosterone boosters, herbal extracts, and others that use proprietary blends [10]. These products are currently the most associated with reports of fraud and deception online [11,12].

Since several contamination cases from supplements and food products have been reported among athletes across various sports disciplines [13], it is crucial to implement rigorous control measures. First, it is essential to assess whether the use of a supplement is necessary, following the FFNFO framework. Verifying the target populations for which supplement classification systems are intended is recommended. Additionally, it is important to choose brands certified by third-party organizations (e.g., NSF, BSCG, Informed-Sport, Cologne List), which verify the identity, quality, and quantity of ingredients. When applicable, these organizations also verify the freshness (for liquid oils) and disintegration (for tablets) of the supplements [14].

Researchers and professionals are encouraged to read the Strategic Plan for 2025–2029 from the Office of Dietary Supplements (ODS) of the National Institutes of Health (NIH) in the United States, as the Dietary Supplements Research Coordinating Committee (DSRCC) has been established to promote collaboration and the coordination of research and training activities on supplements (https://ods.od.nih.gov/About/NIHDSRCC.aspx, accessed on 29 July 2024). In Europe, it is recommended to closely follow the publications and news from the European Food Safety Authority (https://www.efsa.europa.eu/es, accessed on 29 July 2024). Likewise, consumers are encouraged to report adverse effects associated with supplement use on the platforms provided by each governmental regulatory entity (FDA in the US, INVIMA in Colombia, COFEPRIS in Mexico, ANMAT in Argentina, AECOSAN in Spain, etc.). Finally, healthcare professionals and consumers in general can take advantage of free access to original dietary supplement labels to seek products with high-quality raw materials and third-party seals by consulting the NIH dietary supplement label database (https://dsld.od.nih.gov/, accessed on 20 August 2024).

It is crucial to ensure that professional work is based on scientific evidence, and therefore, continuous research in sports nutrition should be promoted to provide practical recommendations for dietary supplements that demonstrate efficacy and safety [15]. As editors of high-impact scientific journals, we invite the scientific community to submit their work in the research areas described in this Editorial, following the recommendations outlined in the PRESENT and PERSiST guidelines as extensions of CONSORT and PRISMA for clinical trials and systematic reviews with meta-analyses in sports nutrition, respectively.
